# Measurement of the absolute value of the optical birefringence of myelin in primate brain tissue

**DOI:** 10.1117/1.NPh.13.2.025011

**Published:** 2026-06-16

**Authors:** Ting Xie, Anna Novoseltseva, Alexander J. Gray, Mikayla Bradsby, Irving J. Bigio

**Affiliations:** a Boston University, Department of Electrical and Computer Engineering, Boston, Massachusetts, United States; b Boston University, Department of Biomedical Engineering, Boston, Massachusetts, United States; c Boston University, Department of Physics, Boston, Massachusetts, United States; d Boston University School of Medicine, Department of Medicine, Boston, Massachusetts, United States

**Keywords:** optical anisotropy, birefringence microscopy, neurodegenerative diseases, myelin quantification

## Abstract

**Significance:**

Researchers require quantitative biomarkers to accurately identify neurodegenerative diseases and quantitatively monitor disease progression. The structural anisotropy of myelin leads to strong optical birefringence, enabling quantitative imaging with polarized-light microscopy imaging for detailed myelin assessment in neurodegenerative disease states and aging studies.

**Aim:**

Our aim is to measure the absolute refractive index difference (birefringence) of the myelin sheath of primate brain tissue using birefringence microscopy (BRM).

**Approach:**

Three-micron cryo-sectioned samples from the paraformaldehyde-fixed corpus callosum of a 22-year-old male rhesus macaque were analyzed using a BRM system. To quantify the absolute birefringence, transversely oriented axons were imaged with a 40× objective under red light-emitting diode illumination (λ=625  nm). Measurements focused on large myelinated axons (diameters ∼2 to 8  μm) mounted in 85% glycerol, ensuring high-resolution characterization of thick myelin sheaths.

**Results:**

The myelin birefringence was determined to be Δn=0.012±0.001, representing the first absolute measurement of this optical property for myelin in primate brain tissue.

**Conclusions:**

This absolute birefringence value enables quantification of myelin volume fraction and assessment of myelin loss in *ex vivo* measurements, providing an accurate optical biomarker for neurodegenerative diseases and aging studies through polarized light microscopic imaging.

## 1 Introduction

The myelin sheath, a bilipid membrane that envelops axons of the central nervous system, serves as a critical electrical insulator, enabling saltatory conduction of action potentials. Its ordered lipid layer arrangement creates structural anisotropy and consequent anisotropy of polarizability, generating strong optical birefringence that can be imaged using polarized light. In neurodegenerative conditions such as Alzheimer’s disease^1^ and multiple sclerosis,^2^^,^^3^ myelin degradation manifests as both loss of myelin and structural disorganization such as loosening, swelling, and fragmentation of the myelin layers. These changes, affecting the thickness and the organization of the myelin sheath’s lipid and protein layers, alter its structural anisotropy and subsequently its birefringence. Quantitative imaging of myelin content and structural integrity could therefore enhance our understanding of these diseases’ progression.

In uniaxial birefringent media, such as myelin, incident polarized light separates into extraordinary (e-ray) and ordinary rays (o-ray), which experience different refractive indices ($n_{e}$ and $n_{o}$, respectively). The true birefringence, defined as the maximum difference in these refractive indices ($\Delta n=|n_{e}-n_{o}|$), is an intrinsic property of anisotropic media. The optic axis characterizes the medium’s birefringence: light propagating parallel to this axis experiences no birefringence, whereas propagation perpendicular to the axis reveals maximum birefringence (Δn) [Fig. 2(b)].

Measuring the relative retardance between ordinary and extraordinary rays propagating through a tissue section of known thickness enables determination of myelin density (volume fraction), contingent on knowing the absolute value of Δn. Thus, accurate measurement of Δn is an important parameter for quantitative imaging of myelin loss and structural degradation in neurodegenerative diseases.

Various polarized light imaging techniques have been employed to study myelin breakdown, including quantitative birefringence microscopy (qBRM),^4^^,^^5^ three-dimensional (3D) polarized light imaging (3D-PLI),^6^^,^^7^ and polarization-sensitive optical coherence tomography (PS-OCT).^8^ Although these methods reveal changes in myelin through relative retardance measurements, some are limited in lower resolution and cannot resolve single-axon structural defects. Fortunately, qBRM offers optical diffraction-limited resolution and has been recently employed by our group to image detailed myelin degradation in neurodegenerative diseases.^5^^,^^9^^,^^10^

Previous studies estimated the true birefringence of myelin to be ∼0.001875^11^ and $6.4\times 10^{-4}$^12^^,^^13^ under the following unrealistic assumptions: (1) that the optic axis of the myelin sheath is parallel to the longitudinal nerve fiber axis, despite the perpendicularity of their orientations; this introduces systematic errors in the calculation of Δn; (2) that all nerve fibers are assumed to lie perfectly parallel to the section plane within the region of interest; and (3) that myelin is assumed to be homogeneously distributed at a 100% volume fraction throughout the tissue sections that were studied (60 and 150  μm).^14^^,^^15^ Generally, the absolute intrinsic value of birefringence (Δn) can be calculated from the measured relative retardance but only if the volume fraction of the myelin is known.

We aim to more accurately determine the value of Δn by taking the optic axis orientation and the volumetric distribution of myelin into account. An early study of frog sciatic axons established an analytical relationship between retardance and true birefringence in myelin of longitudinal fibers (fibers parallel to the section surfaces), yielding a value of ∼0.011.^16^ Although molecular dynamics simulations and spectroscopic studies of lipids and liposomes have provided additional insights,^17^^–^^20^ precise birefringence values for myelin in the mammalian central nervous system remain elusive.

This study presents rigorous measurements of the true (intrinsic) birefringence (Δn) of myelin within the paraformaldehyde-fixed corpus callosum of a 22-year-old male rhesus macaque using a birefringence microscopy (BRM) system. We selected macaques for the close architectural similarity to human brain tissue in axon and myelin sheath structure^21^^,^^22^ and because of the well-controlled post-mortem preservation established by colleagues at the Boston University School of Medicine.^23^^,^^24^ Our findings potentially enable assessment of myelin volume fraction in white matter, offering a quantitative approach to measure myelin loss in advanced neurodegenerative diseases. Although 3D-PLI techniques^15^ and PS-OCT^12^^,^^25^ excel at mapping fiber bundle orientation, they often rely on an assumed or fitted value Δn to derive the inclination angles. Our work complements these techniques by providing a ground-truth experimental value of Δn. Furthermore, the true birefringence value of myelin will enable the simulation of measurement-like BRM images of myelin.

## 2 Methods

### 2.1 Workflow Overview

**Fig. 1:**
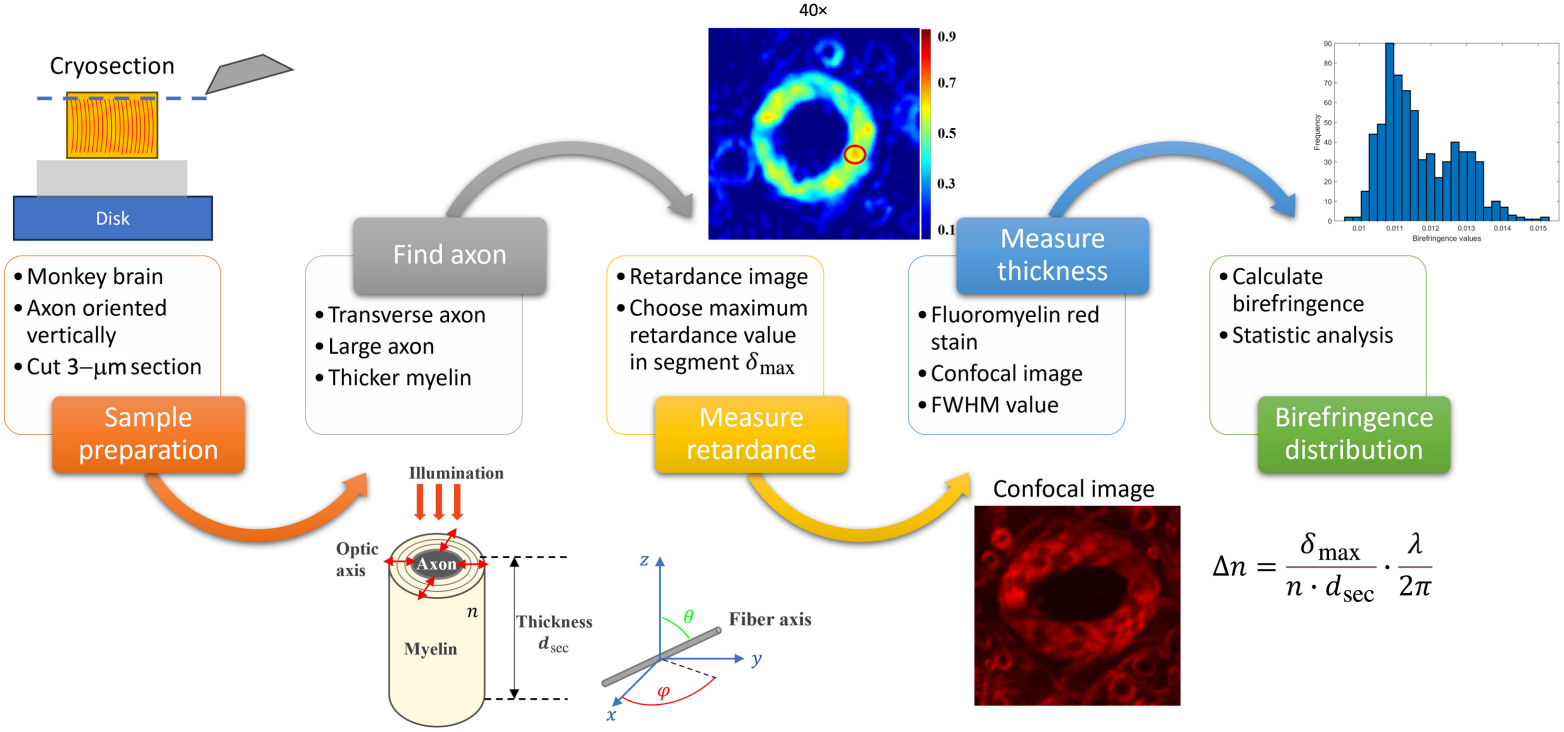
Workflow overview for the true birefringence measurement of myelin sheath.

The birefringence measurement workflow (Fig. 1) integrates several steps: sample preparation, identification of suitable myelinated axons, retardance measurements using qBRM, sample thickness measurement for each myelin segment, and analysis of the birefringence distribution across 720 measured retardance values within 18 myelinated axons. A detailed explanation of each step is provided in Secs. 2.5, 3.1 and 3.3.

### 2.2 Principle

**Fig. 2:**
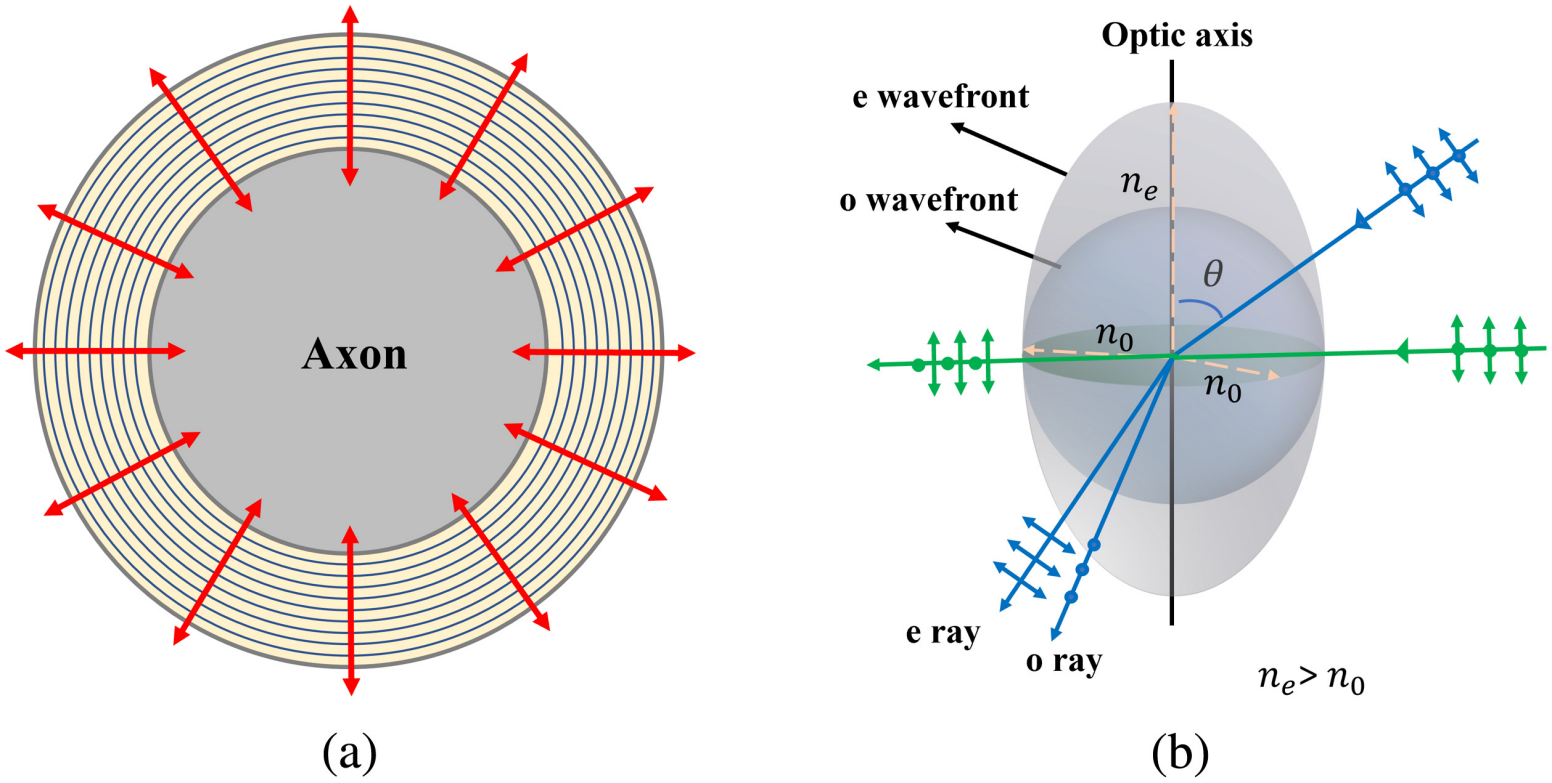
Diagram of (a) myelinated axon and (b) positive uniaxial crystal.^27^

The myelin sheath, which envelopes axons, is a positive uniaxial birefringent medium. This property is due to its double-lipid-layer structure, which induces anisotropic polarizability with respect to the light’s electric field orientation (polarization). The optic axis is oriented normal to the bilayer surface. As a result, for multiple layers wrapped around an axon (typically dozens of layers), the optic axis of the myelin sheath is oriented radially from the center of the axon cross-section [Fig. 2(a)].^26^

As mentioned above, the optic axis in a uniaxial birefringent medium is the propagation direction along which light experiences no birefringence (although biaxial birefringence occurs in some crystals, it does not occur in biological soft tissues). When light propagates through a positive uniaxial material, it splits into two components: the ordinary ray (o-ray), with polarization perpendicular to the optic axis, and the extraordinary ray (e-ray), with polarization parallel to the optic axis. For positive birefringence (as in myelin), the refractive index ($n_{e}$) experienced by the e-ray is smaller than the refractive index ($n_{o}$) experienced by the o-ray. The o-ray experiences a uniform refractive index ($n_{o}$) and follows Snell’s law, propagating uniformly in all directions within the plane perpendicular to the optic axis. In contrast, the e-ray exhibits more complex behavior, as its effective refractive index, $n_{e}(\theta )$, is heterogeneous and varies with the angle (θ) between the propagation direction and the optic axis [Fig. 2(b)].

Light propagation in birefringent media varies with orientation relative to the optic axis.

• Parallel (θ=0  deg): When light propagates parallel to the optic axis, the o-ray and e-ray propagate collinearly and experience an identical refractive index ($n_{o}$).
• Intermediate angles (0  deg<θ<9  deg): As the propagation angle relative to the optic axis increases, the beam progressively splits into distinct o-ray and e-ray components.
• Perpendicular (θ=90  deg): When the incident beam is perpendicular to the optic axis, the o-ray and e-ray again propagate in the same direction; however, they experience the maximum difference in refractive indices, $\Delta n=|n_{e}-n_{0}|$.

where Δn is the true value of the birefringence and is an intrinsic physical property of myelin.

The angle-dependent extraordinary refractive index $n_{e}(\theta )$ leads to the equation^28^

$$\Delta n(\theta )=|n_{e}(\theta )-n_{0}|=\Delta n\cdot \sin^{2}(\theta ),$$ (1)

where Δn(θ) is sometimes referred to as the “apparent birefringence.”^12^^,^^25^ The resulting phase retardance (δ) between the e-ray and o-ray is given by

$$\delta =\frac{2\pi \rho }{\lambda }\cdot \Delta n\cdot \sin^{2}(\theta )=\frac{2\pi }{\lambda }\Delta n\cdot \sin^{2}(\theta )\cdot n\cdot d,$$ (2)

where n is the average refractive index of the myelin sheath, ρ is the average optical path of the e-ray and o-ray within the myelin structure, d is the thickness of myelin, and λ is the vacuum wavelength of the incident light.

### 2.3 Approach

From Eq. (2), we know that the retardance (δ) depends not only on the true birefringence (Δn) of the material but also on the angle (θ) between light propagation and the optic axis and on the optical path length (ρ) within the myelin structure.

**Fig. 3:**
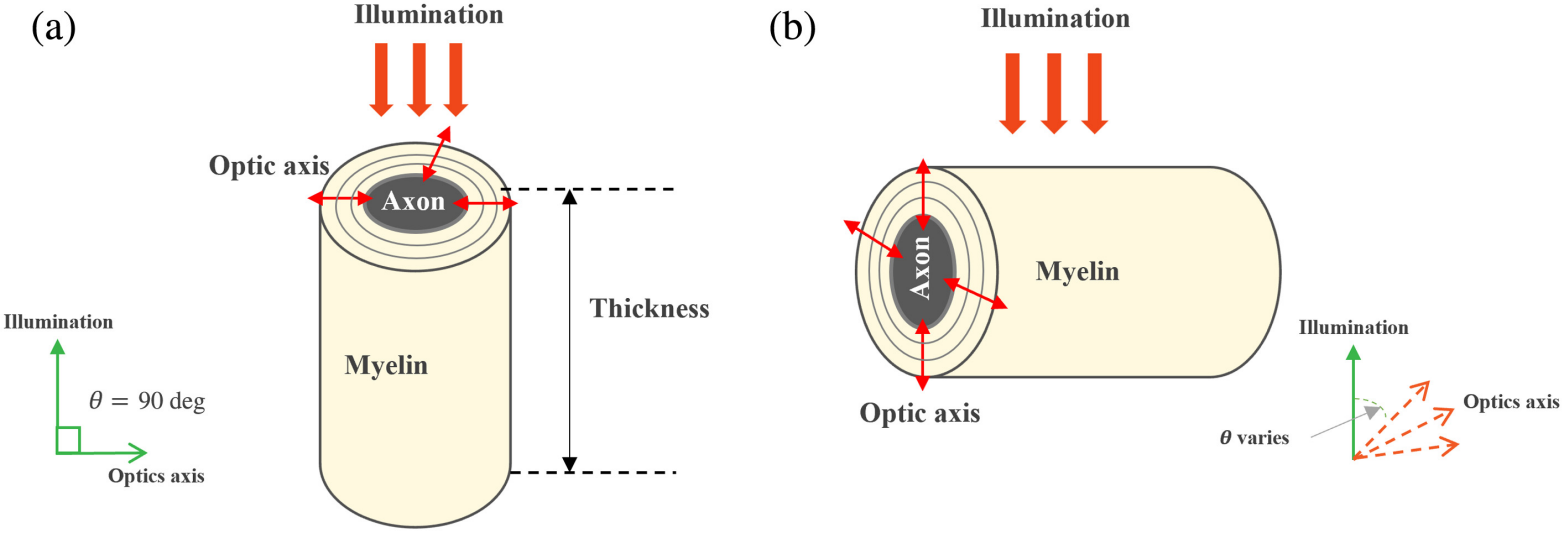
(a) Diagram of the transverse myelinated axon with light propagating perpendicular to its optic axis. (b) Diagram of the longitudinal myelinated axon.

To determine the true birefringence value of myelin, our approach involves selecting large, undamaged myelinated axons that are oriented near-perfectly transverse to the section surface in a thin sample, and then measuring the retardance values. In the case of such transversely oriented fibers, the optic axis of the myelin sheaths is parallel to the section surface and thus perpendicular (θ=90  deg) to the direction of light propagation. As such, the retardance accumulates to its maximum value [Fig. 3(a)]. Under these conditions, the geometric factor $\sin^{2}(\theta )\approx 1$ along the entire section thickness $d_{\sec}$ and Eq. (2) simplifies to

$$\delta =\frac{2\pi }{\lambda }\cdot \Delta n\cdot \rho .$$ (3)

In addition, this approach is justified because the myelin sheath remains continuous between the top and bottom surfaces of the thin tissue section, approximating 100% myelin volume fraction within the focal volume. Consequently, the optical pathlength simplifies to the tissue section thickness multiplied by the sample refractive index ($\rho =n\cdot d_{\sec}$). Thus, the true birefringence can be calculated directly from the retardance values

$$\Delta n=\frac{\delta }{n\cdot d_{\sec}}\cdot \frac{\lambda }{2\pi }.$$ (4)

In contrast, for myelinated axons oriented parallel to the section surface (“longitudinal” axons), the optic axis angle, θ, varies along the light path [Fig. 3(b)], and the myelin structure does not occupy the full light path. Therefore, it would be difficult to measure the true birefringence directly from the measured retardance of “longitudinal” myelin. This is why we chose purely transverse myelinated axons instead of longitudinal myelinated axons or densely packed longitudinal myelin in a thick section (60 or 150  μm)^12^^,^^14^^,^^15^ to determine the true birefringence of myelin.

To obtain transverse myelinated axons and minimize structural variation along the optical path, we prepared thin (3  μm) tissue sections, cut at ∼90  deg to the fiber bundle direction of the corpus callosum. This thickness allows us to approximate the surrounding myelin sheath as a cylindrical structure and ensures the axon’s longitudinal axis is nearly normal to the section surface, for accurate transverse fiber orientation analysis. This thin sectioning also improves imaging clarity in dense white matter regions, which is beneficial for the measurement of inclination angle of myelinated axon fibers, as demonstrated in Sec. 3.1.

### 2.4 BRM System

To measure the intrinsic birefringence of myelin, we first measured the phase retardance utilizing qBRM. qBRM is a label-free imaging technique that visualizes and measures the retardance (δ) and optic axis orientation (φ) of anisotropic materials through their intrinsic birefringence properties.

**Fig. 4:**
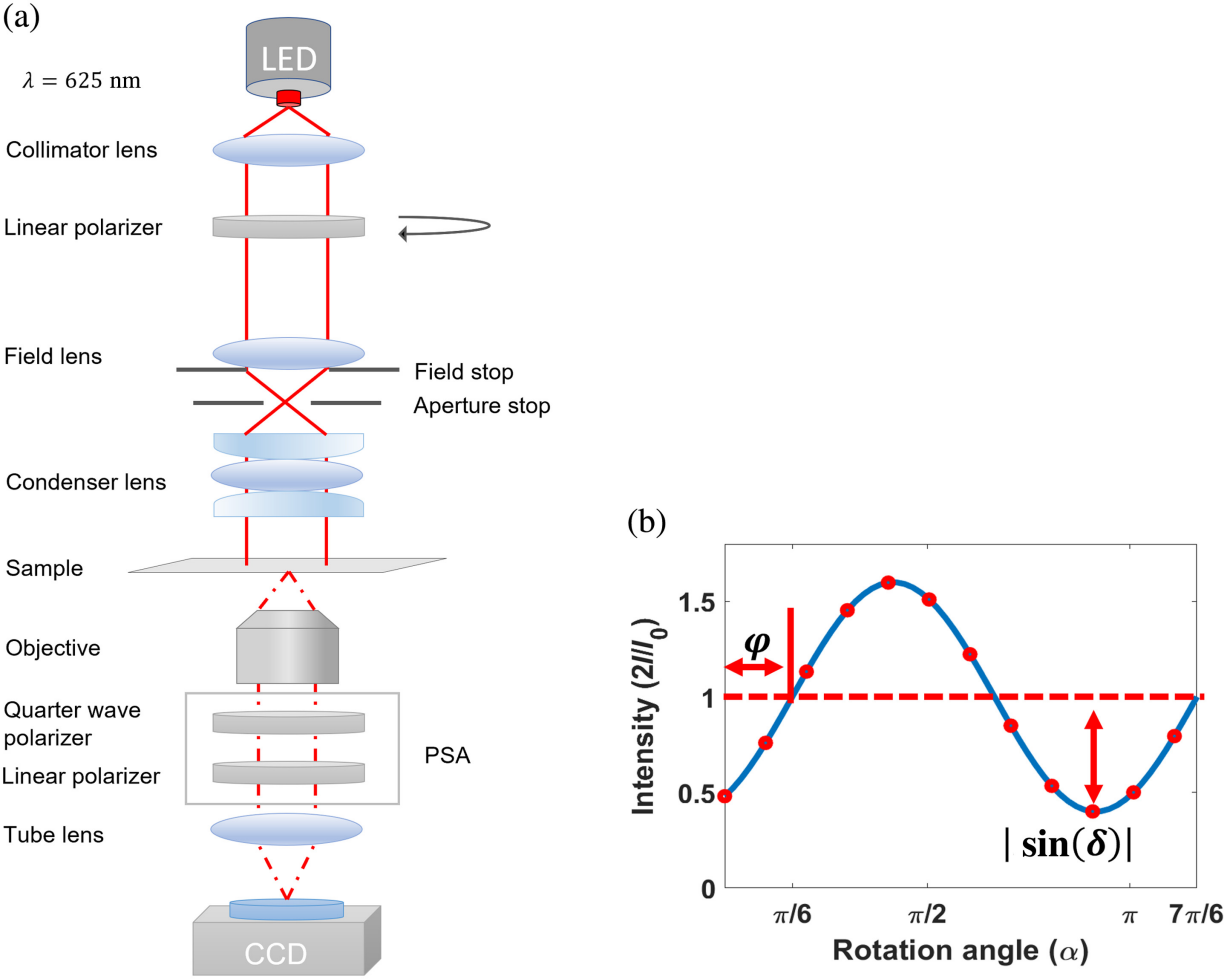
(a) BRM schematic. The system comprises a rotatable linear polarizer for generating linearly polarized illumination, interchangeable objectives (10×/NA=0.3, 20×/NA=0.5, 40×/NA=0.75, and 60×/NA=1.35 oil immersion), a polarization state analyzer with quarter-wave plates and linear polarizers, and a charge-coupled device camera for image acquisition. (b) Intensity I of each pixel in the image plane is a sinusoidal function of the rotation angle α of the polarization of the incident light.

The principle of qBRM involves placing the sample between a rotatable linear polarizer and a circular polarization analyzer [Fig. 4(a)] and capturing images at different rotation angles (α) of the linear polarizer.^29^ The intensity I of each pixel in the image is a sinusoidal function of the rotation angle α of the incident light’s polarization [Fig. 4(b)]

$$I=\frac{I_{0}}{2}[1+\sin(2\alpha -2\varphi )\cdot \sin(\delta )],$$ (5)

where $I_{0}$ is the pixel-specific intensity of the illumination light, φ represents the orientation of the optic axis projection on the image plane, and δ is the phase retardance.

In our implementation of qBRM, by imaging at three or more angles, α, of the linear polarizer and capturing the corresponding intensities I, we can extract the three unknown parameters, $I_{0}$, φ, and δ, for each pixel.^5^ Given the relation between retardance and birefringence in Eq. (4), the birefringence value of the sample can be calculated from the measured relative retardance, sin(δ).

Aperture stops, positioned at the focal planes of both the field lens and condenser lens, control the illumination numerical aperture and, consequently, the angular distribution of the illuminating light cone. The range of the illumination angle directly influences the measured retardance values, as the optical path difference experienced by polarized light varies with the incident angle through the birefringent sample.

### 2.5 Tissue Preparation

A block of rhesus macaque brain tissue from the corpus callosum, fixed in 4% paraformaldehyde for at least 24 h post-harvest (with post-mortem interval of a few minutes), was obtained from collaborators at the Boston University Medical Center. The sample was taken from a rhesus macaque (22-year-old male) with a cortical injury area far from the corpus callosum. We chose primate (monkey) brain tissue because it is the closest analog to human brain tissue and was carefully preserved, with no significant post-mortem interval, a common confounding factor with available human brain tissue. The motivation for choosing the corpus callosum was simply because of its consistent axon orientation.

A smaller block (around 4  mm×4  mm×4  mm) of tissue was cut with a knife, ensuring the surface normal was aligned with the predominant orientation of the fiber bundles, so that most axons were oriented transversely to the surface of the section (sucrose was used to cryo-protect the tissue block, and the block was fast-frozen). The tissue block was then cryo-sectioned into 3-μm sections using a Leica CM1950 Cryostat microtome^29^ and then mounted in 85% glycerol. Sucrose and paraformaldehyde were replaced by 85% glycerol during the mounting process. Glycerol and water are not birefringent, and we submit that they would not affect our measurements. Details of the cryosectioning process are provided in Sec. 1 in the Supplementary Material.

No animals were euthanized for this study, as we used tissue that had been previously preserved and archived for other (NIH-funded) studies (care of the animals used in this study was accredited by the Association for the Assessment and Accreditation of Laboratory Animal Care and in accordance with the Guide for the Care and Use of Laboratory Animals from the National Institutes of Health’s Office of Laboratory Animal Welfare and approved by the Institutional Animal Care and Use Committee of Boston University Medical Campus).

## 3 Results

### 3.1 Images of Transverse Myelin

Several 3-μm brain sections containing transversely oriented myelinated axons were imaged at 40× magnification (NA=0.75, resolution∼400  nm) using qBRM to quantify retardance in the myelin segments.^26^ We acquired z-stacks of qBRM retardance maps and retardance-weighted optic axis orientation maps of brain sections at depth intervals of 1  μm throughout the 3-μm section thickness, thus acquiring 10 planes for each tissue section to account for potential non-flatness (the focal planes started a few microns above and ended a few microns below the section, thus assuring that the automated system always captured a focal plane at or near the center of the tissue section).

**Fig. 5:**
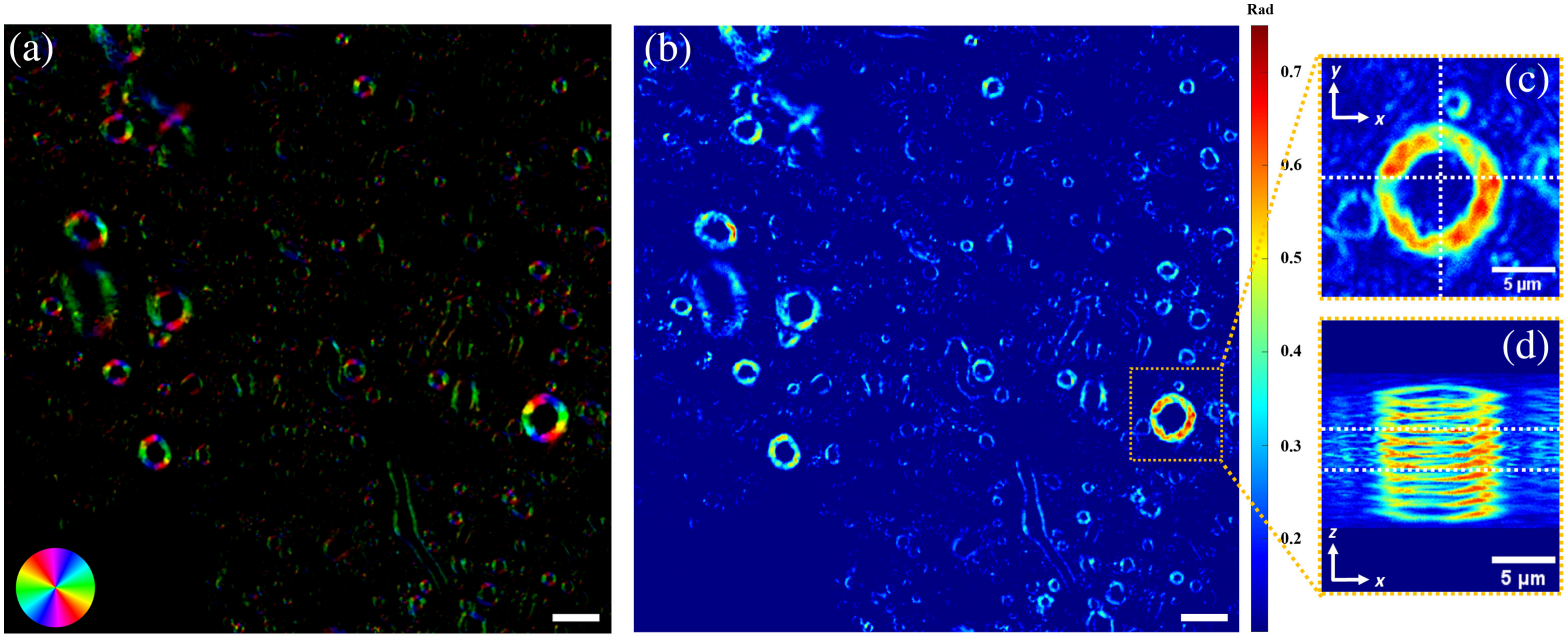
(a) qBRM image of transverse myelin using an objective at NA=0.75, with color encoding the orientation of the optic axis (match with color wheel) in the myelin sheath and brightness indicating the relative retardance value. (b) Corresponding relative retardance map. Scale bar: 10  μm. (c) and (d) Zoomed-in lateral and axial views of one transverse myelin segment; the actual myelin segment is located between two white dash line in panel (d); the inclination angle of the fiber axis is close to 90 deg.

Figures 5(a) and 5(b) show the orientation and retardance maps of the middle plane containing transversely oriented myelin segments. In Fig. 5(a), the color encodes the projected orientation of the optic axis (corresponding to the color wheel), and the brightness indicates the relative retardance value. In Fig. 5(b), colors constitute a “heat map” representation of the retardance value, with red indicating high retardance and blue indicating low retardance. As expected from the theoretical discussion in Sec. 2.3, the transversely oriented myelin exhibits higher retardance than longitudinally oriented myelin.

Optimally oriented transverse myelin segments were identified by their stationary position across different z-planes, as demonstrated in the zoomed-in lateral and axial views of one myelin segment in Figs. 5(c) and 5(d). In Fig. 5(d), the actual myelin segment is located between the two white dashed lines. We measured the fiber axis inclination angle within the dashed lines to be close to 90 deg with respect to the section plane. Accordingly, we selected transverse myelin segments that exhibited a 90-deg inclination angle for the subsequent birefringence measurements in Sec. 3.2. Most transverse myelin segments display the characteristic ring structures in Fig. 5 due to their radially oriented optic axes lying in the section plane. Variations in retardance values among transverse myelin segments are attributed to structural imperfections in the myelin sheath or slight deviations from a perfectly transverse orientation.

### 3.2 Dependence of Measured Retardance on Objective Numerical Aperture (NA)

In experiments measuring the relative retardance of myelin, we observed that the values obtained for the same segments varied depending on the NA of the objective lens used. To investigate this dependence, we imaged transversely oriented myelin segments using microscope objectives with different NAs (0.3, 0.5, 0.75, and 1.35, the latter with oil immersion).

**Fig. 6:**
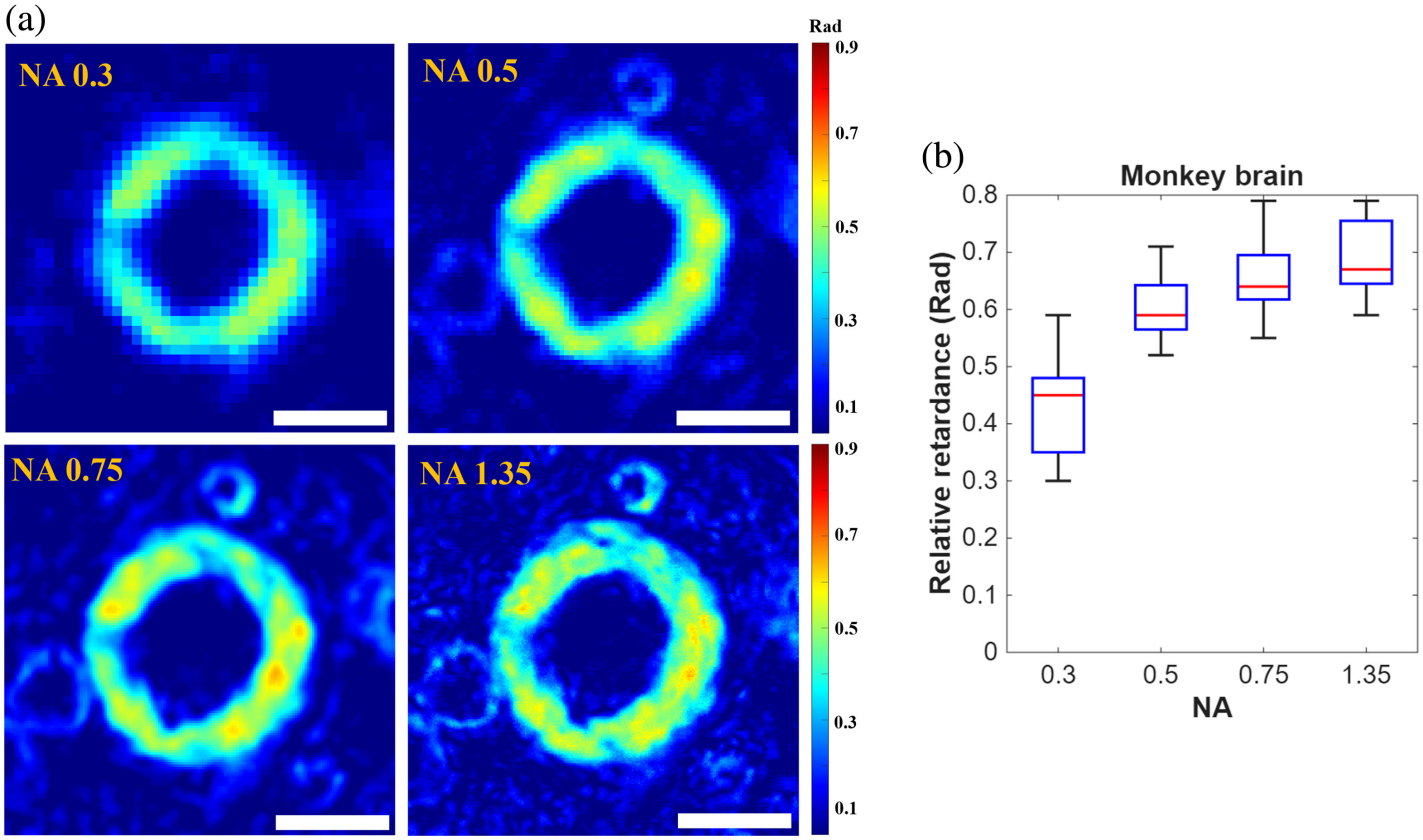
(a) Relative retardance maps of a representative myelin segment were acquired using microscope objectives with NAs of 0.3, 0.5, 0.75, and 1.35 (the latter employing oil immersion), scale bar: 5  μm. (b) Quantitative analysis of transverse myelin segments (n=11 retardance measurements from 11 distinct myelin segments) showing box plots of maximum retardance. The measured retardance value systematically increases with NA due to resolution effects.

The relative retardance (sin(δ)) maps of a representative transverse axon for each NA are shown in Fig. 6(a). Higher magnification objectives (i.e., NA=0.75 or 1.35) revealed finer structural details. Maximum retardance values for transverse myelin segments were extracted from z-stacked retardance maps using MATLAB and FIJI, with the distribution across magnifications shown in Fig. 6(b).

We specifically chose segments with larger axons and thicker myelin sheaths to ensure that light transmission was continuously within the myelin and to minimize measurement variability. In addition, larger structures occupy more pixels on the detector, thereby improving the signal-to-noise ratio of retardance measurements. We also explored the invariance of myelin optical properties across a range of axonal diameters, presented in Sec. 6 in the Supplementary Material.

As shown in Fig. 6(b), the box plots indicate that the measured relative retardance values increase markedly between NA=0.3 and NA=0.5, followed by a plateau between NA=0.75 and NA=1.35. The difference in retardance values between NA=0.75 and NA=1.35 was not statistically significant, suggesting that the measurements approach an asymptotic value, approximately sin(δ)=0.65.

The variation in measured relative retardance with NAs can be attributed to the linear incoherent imaging system (BRM system), where the objective functions as a low-pass filter.^30^ Higher NA objectives provide superior lateral resolution, which is essential for resolving the thin myelin ring of smaller axons.

The observed “drop” in measured retardance at lower NAs [Fig. 6(b)] is an understood optical effect resulting from the resolution spot size exceeding the myelin wall thickness, thereby averaging the birefringent signal from the myelin with the non-birefringent background. Furthermore, objectives with lower NA have lower cutoff spatial frequencies, leading to the loss of high-frequency components during image formation. This loss results in smoothing of high spatial-frequency features (pixels with high retardance values), thus averaging high- and low- retardance regions. A detailed analysis of the effects of the cutoff frequency is provided in Sec. 2 in the Supplementary Material.

To address other potential sources of retardance variation, we also demonstrated the impact of the condenser lens NA on the retardance measurement in Sec. 3 in the Supplementary Material. This analysis showed that the angular variation introduced by the condenser lens only causes a minor perturbation in the measured retardance.

Consequently, objectives with high NA provide superior spatial frequency response and therefore yield retardance measurements that more accurately reflect the intrinsic birefringence of the myelin. The similarity in relative retardance values obtained using the NA=0.75 and NA=1.35 objectives [Fig. 6(b)] helped justify our selection of the NA=0.75 objective for the subsequent quantitative analysis for determination of the intrinsic value of the myelin sheath birefringence.

### 3.3 Determination of the True Birefringence of Myelin

**Fig. 7:**
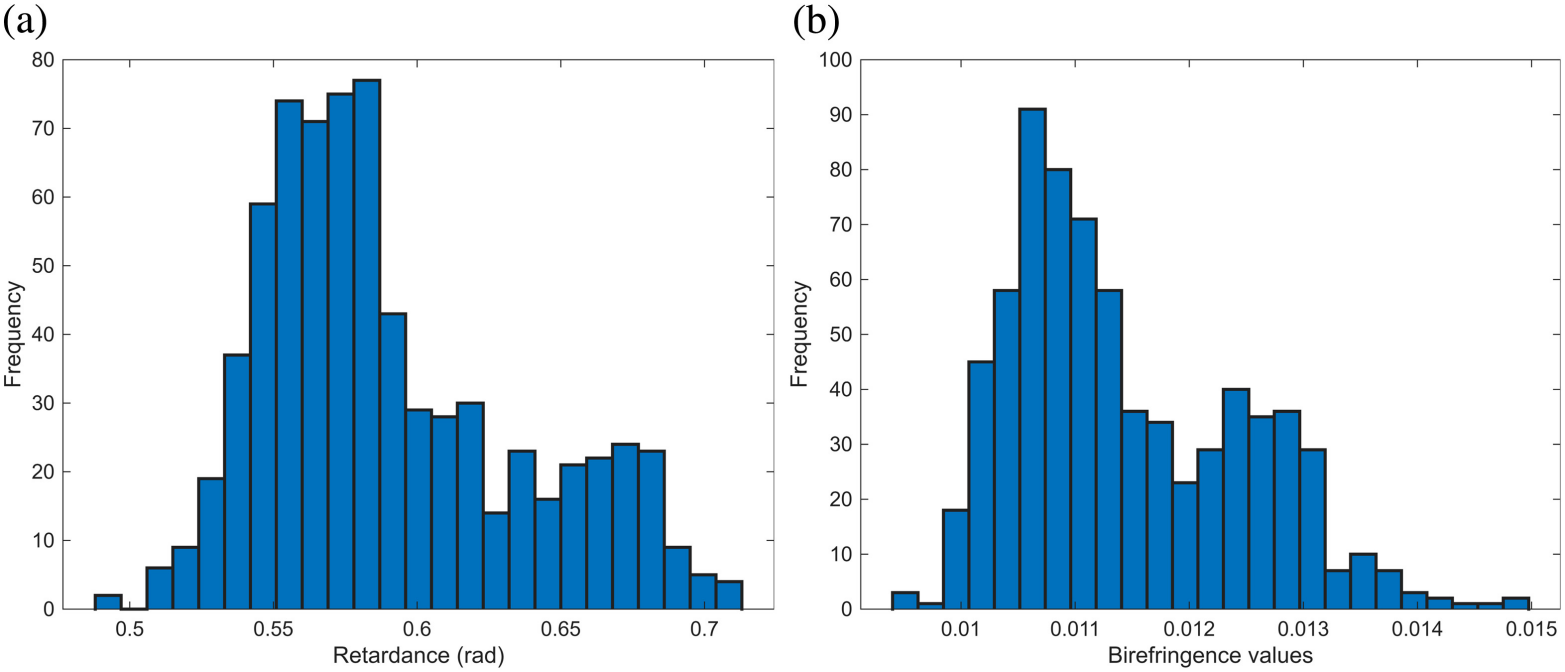
Statistical analysis of myelin retardance and birefringence (n=45 retardance measurements from 18 distinct myelin segments). (a) Histogram showing the distribution of retardance values measured from multiple myelin segments. (b) Corresponding histogram of calculated birefringence values from the same myelin segments.

Subsequently, we selected the maximum retardance value within each myelin segment to represent the segment’s retardance. To minimize the noise inherent in individual peak values, we identified local retardance maxima, qualitatively observed as high-intensity regions across multiple focal planes. For each identified maximum, retardance was measured for all pixels within a 4×4 window centered on the peak. By sampling 45 such windows across 18 myelinated axons (capturing multiple windows per axon), we generated a distribution of 720 retardance values, as illustrated in Fig. 7(a).

We selected the maximum retardance value for several critical reasons: (1) Partial volume mitigation deriving from pixels at myelin ring edges that contain a mixture of myelin and background, artificially lowering the measured retardance, only pixels for which the optical path fully traverses the myelin sheath provide the true material property. (2) Volume fraction maximization of myelin (∼100%). The maximum values represent locations where myelin occupies the full optical pathlength, satisfying $\Delta n=\delta_{\max}\cdot (\lambda /2\pi )/(n\cdot d_{\sec})$, whereas averaging over the ring would systematically underestimate Δn. (3) Addressing structural irregularities: the maximum value corresponds to the most intact, well-organized regions, providing the closest representation of the true intrinsic birefringence despite local variations in thickness or packing density. (4) Focus optimization: maximum retardance ensures sampling of in-focus portions despite finite depth of field. This approach parallels the standard practice of using the brightest pixel in fluorescence microscopy to measure peak concentrations.^31^

Given the derived retardance, we can calculate the birefringence value (Δn) of myelin sheath using Eq. (4), provided the actual thickness of each myelin segment is known. Although the cryostat microtome was set for a nominal section thickness of 3  μm, the actual section thickness varies due to mechanical precision limitations, temperature fluctuations, and sample mounting artifacts. Precise thickness determinations were obtained using confocal microscopy of FluoroMyelin-Red-stained samples^32^ (see Fig. S4 in the Supplementary Material). Figure 7(b) shows the birefringence distribution incorporating the measured thickness values for each segment.

Finally, statistical analysis resulted in a value for the myelin sheath birefringence of Δn=0.012±0.001 in the rhesus macaque brain. Importantly, a *post hoc* analysis of the 18 measured axons confirmed that birefringence and retardance values exhibit no systematic dependence on the axonal diameter or the thickness of myelin sheath in transversely myelinated axons for the range of axon diameters measured (2 to 8  μm) (see Fig. S5 in the Supplementary Material). This is consistent with the view that birefringence is an intrinsic material property of the myelin lipid bilayer architecture, rather than a function of macroscopic morphology. The detailed differences between the distributions of retardance and birefringence (Fig. 7) are primarily due to the small variations in myelin sheath thickness across different segments, which translate to differences in effective optical pathlength ρ in Eq. (4) (if all myelin segments had the same thickness, the birefringence distribution would better match the retardance distribution). However, the variation of thickness measurements is also influenced by the confocal microscope’s limited axial resolution. In addition, the appearance of two noisy peaks in the retardance and birefringence distributions may be attributed to imperfect transverse alignment of myelin segments when scanning through z-planes. Ideally, highly retardant regions (red spots) should remain stationary when scanning through different z-planes. In practice, small shifts or changes were observed in these red spots at different z-planes, which can affect statistical analyses by adding noise to the determined retardance values. In short, axons are not perfect cylinders.

## 4 Discussion

In this study, we present an optimized method for measuring the absolute value of the intrinsic birefringence of the mammalian myelin sheath, applied here for rhesus macaque brain tissue. Our measurements determined the birefringence of myelin to be Δn≈0.012±0.001. This determination relied on the assumptions of 100% volume fraction of myelin through the entire section thickness (∼3  μm) and, for this geometry, on the negligible effect of angular variation of light rays traversing the myelin within the focal region.

The value of obtaining the absolute intrinsic birefringence value, Δn, is substantial. It enables quantification of the myelin volume fraction (if the pathlength is known), based on retardance images, thereby allowing for a quantitative assessment of myelin loss. Such information can contribute to the study of neurodegenerative diseases and age-related changes.

Furthermore, the extraction of the phase retardance δ in modalities such as qBRM and 3D-PLI relies on the measured value of |sin(δ)|. This introduces a theoretical ambiguity if δ exceeds π or π/2 (i.e., phase wrapping). Knowing the absolute value of Δn allows for the unambiguous determination of the phase retardance δ using Eq. (3), eliminating the need for more complex multiwavelength approaches to resolve such ambiguities.^33^ This was, however, not an issue for our thin tissue sections, where the measured retardance values were small.

In addition, when both the true birefringence (Δn) and sample thickness are known, the inclination angle (θ) of the myelin sheath can potentially be calculated directly from the retardance values. Another promising application of precise Δn measurement is enabling the development of more accurate computational simulation models for synthesizing measurement-like images of complex myelin fiber structures in brain tissue.^34^ By comparing these simulations with experimental qBRM images of brain tissues, we can better understand the measured signal from fiber mixtures (due to the partial-volume effect), and potentially uncover the intrinsic structure of complex fiber bundles.

Several limitations of the present study warrant consideration. Specifically, all tissue sections were derived from a single rhesus macaque specimen, with measurements restricted to the corpus callosum. Consequently, the reported Δn=0.012±0.001 reflects within-subject measurement variability and does not account for inter-subject biological diversity. Although myelination patterns (e.g., g-ratio, sheath thickness, and molecular composition) can vary across individuals and tracts, the fundamental architecture of myelin remains highly conserved.^16^^,^^35^^–^^37^ This lipid-rich, multilamellar structure, supported by MBP/PLP scaffolding and consistent optical anisotropy across vertebrate species, suggests that Δn is unlikely to vary substantially across regions or species.^38^ Nevertheless, future multi-region and multi-subject studies, particularly in humans, are necessary to provide empirical confirmation. Furthermore, the current study was performed at 625 nm, and we do not make assumptions about the value of Δn at other wavelengths. That said, as both ordinary and extraordinary axes experience normal dispersion throughout the visible to NIR region, we offer the conjecture that the wavelength dependence of the difference in index, the birefringence, will be small across that range. This, however, remains to be measured experimentally for future work.

Backscattering polarimetric modalities, such as PS-OCT, typically operate in a double-pass geometry, which doubles the accumulated retardance compared with transmission modes. Although determining the precise path length through myelin is more challenging in these configurations, the absolute Δn reported here provides a critical constraint. When combined with structural or model-based path length estimates, this value enables the quantitative interpretation of backscattering retardance and serves as a robust fixed parameter for forward-scattering models.

## Supplementary Material

10.1117/1.NPh.13.2.025011.s01

## Data Availability

All code used for the acquisition and analysis of qBRM data can be found at https://github.com/Ting24k/Myelin-Birefringence-measurement.git. All imaging data are available at the BioImage Archive under accession S-BSST2953: https://www.ebi.ac.uk/biostudies/studies/S-BSST2953.
