# Supplementary material for: Measurement of the absolute value of the optical birefringence of myelin in primate brain tissue
Source: Neurophotonics. 2026 Jun 16;13(2):025011. doi: 10.1117/1.NPh.13.2.025011 (PMC13276737; doi:10.1117/1.NPh.13.2.025011)
Supplement: Supplementary file 1 [file NPh_013_025011_SD001.pdf]

Supplemental material

1 Cryosection process

Before cryo-sectioning the tissue block into 3-μm sections using the Leica CM1950 Cryostat microtome, the block was immersed in a solution of 30% sucrose in phosphate buffered saline (PBS) for cryoprotection. This solution, consisting of 15 g sucrose powder in 50 ml of 1X PBS, was used to accelerate sucrose perfusion. The sample was placed on a platform shaker in a cold room at 4°C and shaken for several days; it was ready for cryo-sectioning once the sample sank.

The entire cryosection process is summarized in Fig. S1. During the process:

1) A 3D-printed hollow cylinder mold was placed onto specimen discs, filled with liquid Optimal Cutting Temperature (OCT) compound, and frozen within the cryostat chamber.
2) The frozen OCT was then removed from the mold but remained adhered to the disk. The disk with the frozen OCT was mounted onto the cutting stage with notch alignment, and a blade was used to cut a flat surface. The notch alignment is to set the specimen chuck and blade holder perfectly parallel, ensuring optimal, consistent sectioning
3) The tissue block was placed onto this flattened OCT surface, ensuring the fiber bundle (indicated by the red line in Fig. S1) was normal to the surface. (The cut surface of the tissue faced down toward the OCT.)
4) The mold was placed back onto the frozen OCT surface, enclosing the tissue block, and filled with liquid OCT to cover the sample. This combined assembly was frozen within the cryostat chamber again.
5) Once the OCT was sufficiently frozen, the mold was removed, and cutting commenced. Initially, a thick cut (60-μm - 100-μm) was performed to remove the extra frozen OCT

above the tissue. As the blade neared the tissue, the cutting steps were gradually reduced to 3-µm.

6) Several 3-µm sections were cut before collecting sections to ensure the thickness had stabilized. The 3-µm sections were then collected. Initially, the cut section was curled; a soft brush was used to gently unfold the section before quickly adhering it to the slide when fully flat.

7) Finally, the sections were then mounted in 85% glycerol ($n \approx 1.47$) with 0.01% sodium azide to optimize refractive-index matching to the myelin ($n \approx 1.47$), thereby reducing scattering in the images.

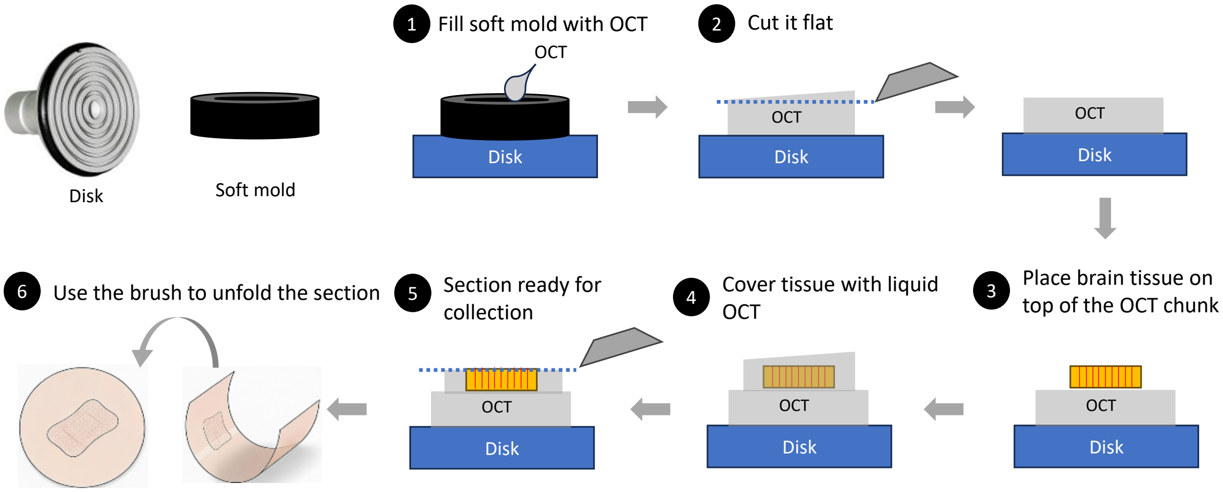

Fig. S1 Cryosection process

2 The effect of microscope objective NA on retardance value

In an incoherent optical microscopy system, the objective lens functions as a low-pass filter, allowing low-frequency components of the object to pass through. The cutoff spatial frequency of an incoherent system is given by $2NA/\lambda$, which is twice that of a coherent system, where NA is numerical aperture of the objective and λ is the illumination wavelength.

An incoherent system is linear with respect to intensity. The relationship between the image intensity I_{image} and the object intensity I_{object} is described by the convolution equation:

$$I_{image}(x, y) = |h(x, y)|^2 \otimes I_{object}(x, y) \qquad (S1)$$

where $h(x, y)$ is the coherent point spread function.

In the Fourier domain, the image intensity is related to the object intensity by:

$$\Theta_{incoh_{image}}(x, y) = \mathcal{H}_{incoh}(x, y) \cdot \Theta_{incoh_{object}}(x, y) \qquad (S2)$$

where $\mathcal{H}_{incoh}(x, y)$ is the incoherent transfer function.

To simulate the effect of a lower NA on image formation (low-pass filtering), we would typically apply this process to the object intensity. However, we cannot directly transform the retardance image because the retardance map in the qBRM system is extracted from three raw images captured under different rotation angles of the linear polarizer. Therefore, we must apply the low-pass filter process and down-sampling separately to the three raw images acquired with the NA = 0.75 objective. The simulated retardance image (for NA = 0.3) is then extracted from these processed raw images.

The results are presented in Fig. S2. Figure S2(a) and S2(b), showing the experimental retardance maps of the same myelin segment acquired using objectives with NA = 0.3 and NA = 0.75, respectively. Figure S2(c) shows the simulated retardance map (for NA = 0.3) after applying a low-pass filter and down-sampling of the NA = 0.75 experimental retardance map.

The strong similarity in retardance distributions between Figs. S2(a) and S2(c) confirms that the lower retardance values observed at lower NAs are caused by the corresponding lower cut-off frequency. This result supports the conclusion that higher-NA objectives provide retardance measurements that more accurately represent the true intrinsic values of myelin.

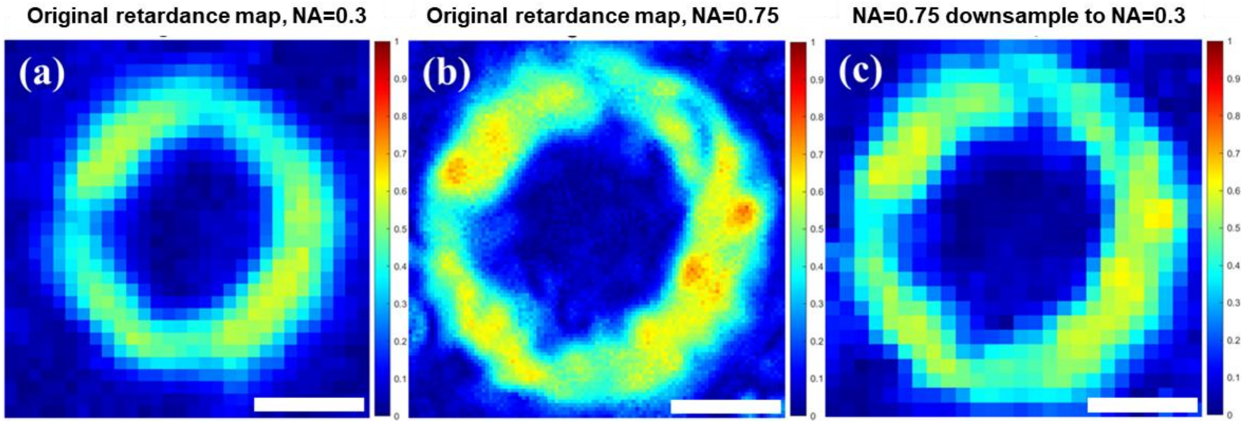

Fig. S2 (a) Original retardance image obtained from an objective with NA = 0.3. (b) Original retardance image obtained from an objective with NA = 0.75. (c) Simulated retardance image (NA=0.3) obtained from the original retardance image (b). The retardance value distributions in (a) and (c) appear similar. The scale bar is 5 μm.

3 Impact of the NA of the condenser lens on the value of the retardance

The numerical aperture (NA) of the condenser lens controls the angle of the illuminating light cone that impinges on the sample. As shown in Fig. S3, the blue cone represents the light cone after the condenser lens, with the maximum angle of the incident light, denoted as θ_0, being 48° ($sin^{-1}(NA_{condense} = 0.75)$).

Based on Snell's Law, when light transitions from air into the sample coverslip, the maximum angle θ_1 is 30°. In our experiment, we used 85% Glycerol (n_1 = 1.47) as the mounting solution, achieving approximate refractive-index matching with the myelin (n_2 = 1.47). Therefore, light propagates straight into the myelin sheath without refraction, implying that the light cone entering the myelin has a maximum solid angle defined by θ_{1_max} =30°. (The optic axis of myelin is oriented horizontally, and the thickness is h.)

By a modification of Eq. (2), the phase retardance δ for oblique incidence is given by:

$$\delta = \frac{2\pi}{\lambda} \cdot \rho \cdot \Delta n \cdot cos^2(\theta_1), \qquad \theta_1 \in [0^\circ, 30^\circ] \qquad (S3)$$

where $\rho = n_2 h / cos(\theta_1)$ represents the optical path length within the myelin. Simplifying the retardance equation gives:

$$\delta = \frac{2\pi}{\lambda} \cdot n_2 h \cdot \Delta n \cdot cos(\theta_1), \qquad \theta_1 \in [0^{\circ}, 30^{\circ}] \tag{S4}$$

To account for the angular distribution of the incident light, we calculate the average value of $cos(\theta_1)$ over the solid angle. The normalized value $\langle cos(\theta_1) \rangle_{norm}$ is computed as:

$$\langle cos(\theta_1) \rangle_{norm} = \frac{\int_0^{2\pi} \int_0^{\theta_{1\_max}} cos\theta \cdot sin\theta \cdot d\theta d\varphi}{\int_0^{2\pi} \int_0^{\theta_{1\_max}} sin\theta \cdot d\theta d\varphi} = \frac{1}{2}\{1 + \cos(\theta_{1\_max})\} \tag{S5}$$

Since $\theta_{1\_max} = 30^{\circ}$, the value of $\langle cos(\theta_1) \rangle_{norm}$ is calculated to be 0.93. The average retardance $\langle \delta \rangle$ is thus expressed as:

$$\langle \delta \rangle = \frac{2\pi}{\lambda} \cdot n_2 h \cdot \Delta n \cdot 0.93 \tag{S6}$$

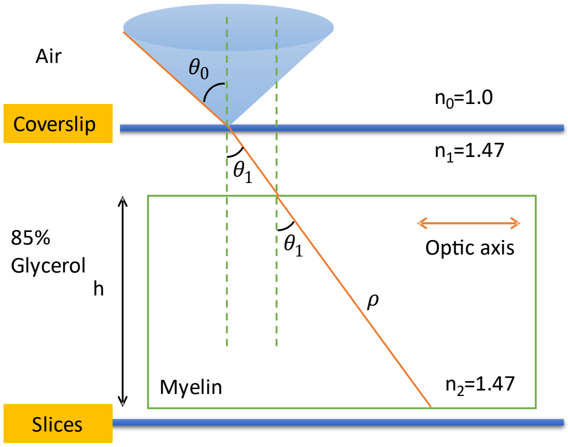

Fig. S3 The diagram of light cone center coverslip and myelin.

The result indicates that the average retardance is 7% lower compared to the case of normal light incidence ($\theta_1 = 0^{\circ}$). This change represents a minor perturbation in the measured retardance compared with the effect of objective's NA, especially because the intensity of the illumination light tapers off towards the edges (highest angles) of the cone.

4 Method to measure the thickness of thin sample

While our cryostat's minimum specified cutting thickness is 3-μm, the actual section thickness can vary due to mechanical precision limitations, temperature fluctuations, and sample mounting artifacts. To determine the precise sample thickness for birefringence calculations, we employed a Nikon CSU-W1 SoRa Spinning Disk Confocal Microscope to a measure stained sample.

The experimental procedure was as follows:

1) First, the mounted 3-μm sections were imaged using the qBRM system to extract retardance maps and identified regions of interest (ROIs) containing proper, continuous myelin segments.
2) Subsequently, the same sections were stained with FloroMyelin^{TM} red (excitation/emission: 558/654 nm) following the protocol discussed in main text. This fluorescent dye specifically labels myelin sheaths through lipophilic interactions with their lipid bilayer structures.
3) The staining process was found not to change the structure or retardance of the myelin, enabling retardance measurement both before and after staining.
4) We then located seven ROIs in one section under the confocal microscope and acquired z-stack images, scanning from the point where the signal first appeared until it disappeared.

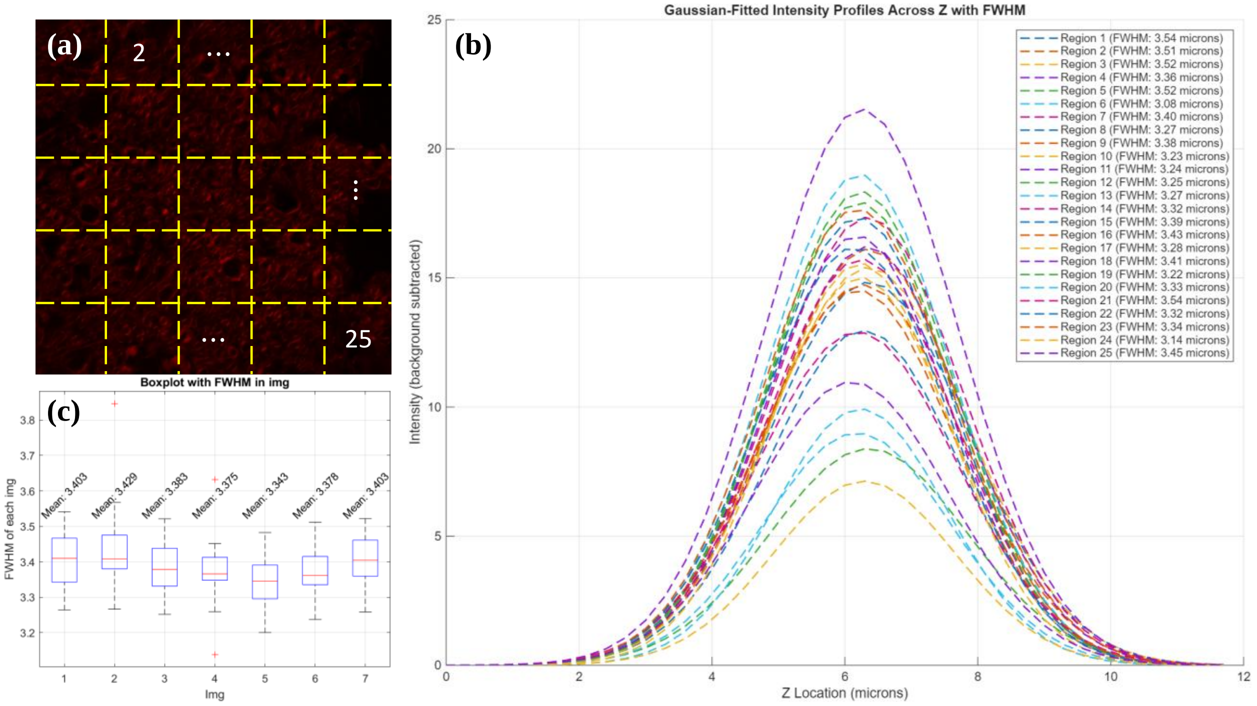

Fig. S4 (a) Diagram of the method to crop the z-stack confocal image into 5*5 regions. (b) Plot of the gaussian fitting curves after subtracting the background for each region in one z-stack image. Each curve represents the average intensity within region over the z plane, enumerate the FWHM of each curve as well. (c) Boxplot of 25 the FWHM in each image, with the mean value of each boxplot.

Figure S4 shows the method we used to measure the thickness of the identified ROIs. We took seven z-stack confocal images at increments of 0.3 μm, covering a 12-μm depth across seven ROIs distributed in one section. Since the scan depth exceeded the sample thickness, the signal started from dark, peaked in intensity at the middle plane, and ended as dark.

For quantitative analysis, these seven confocal images were processed using MATLAB as follows.

1) Each image was divided into 5×5 regions (Fig. S4(a)).
2) The average intensity within each region was calculated at each plane.
3) The resulting intensity profiles exhibited Gaussian-like distributions.

4) After background subtraction and Gaussian fitting, the full-width-at-half-maximum (FWHM) was calculated as representative of the sample thickness for each region. The thickness varied from 3.08-μm to 3.54-μm, confirming that actual section thicknesses often exceeded the nominal 3-μm setting and varied across different regions (Fid. S4(b)).
5) This analysis was performed across seven ROIs, generating 25 FWHM values per ROI. Box plots were generated to represent the distribution (Fig. S4(c)).
6) The results showed consistent average FWHM values of approximately 3.3-3.4 μm across all images, which represents a reasonable deviation from the target thickness.

Finally, this analysis was performed to measure the thickness value for each segment, which was used in the birefringence calculations.

5 Calibration of retardance measurements

To calibrate the retardance measurements of the qBRM system, a zero-order quarter-wave plate (Thorlabs WPQ10ME-633) was employed as a calibration phantom. Assuming a nominal relative retardance of $\pi/2$ for the wave plate, the qBRM-derived retardance was determined to be accurate to within ±5%.

6 Invariance of myelin optical properties across axonal geometries

While myelin thickness and the number of wraps (quantified by the g-ratio) scale with axon diameter, the microscopic organizational structure of its constituent lipids and proteins, and thus its material properties, is expected to vary only minimally, as discussed in the **main text**. Although the specific lipid and protein composition of myelin differs measurably across brain regions and tracts, its **fundamental architecture** (a lipid-rich multilamellar membrane supported by MBP/PLP scaffolding) remains highly conserved. Furthermore, the **optical anisotropy** of myelin

is essentially preserved across species (see **main manuscript for full details and references)**. Consequently, despite minor biochemical variations, differences in the refractive index are small, rendering our measured birefringence value (Δn) a reliable representative for other brain regions and species.

The consistency of birefringence observed across 18 measured axons, spanning a diverse range of diameters and myelin thicknesses (Fig. S5), further confirms that this value is not an artifact of axon size or local microenvironment. For these 18 large axons, diameters ranged from approximately 2 to 8 μm, with myelin thicknesses between 1 and 3 μm. A post-hoc analysis of birefringence as a function of axonal geometry (Fig. S5) revealed no systematic dependence on diameter or thickness for transversely oriented axons. These results confirm that birefringence is an intrinsic material property of the myelin lipid-bilayer architecture, largely independent of axonal caliber or the number of lamellar wraps.

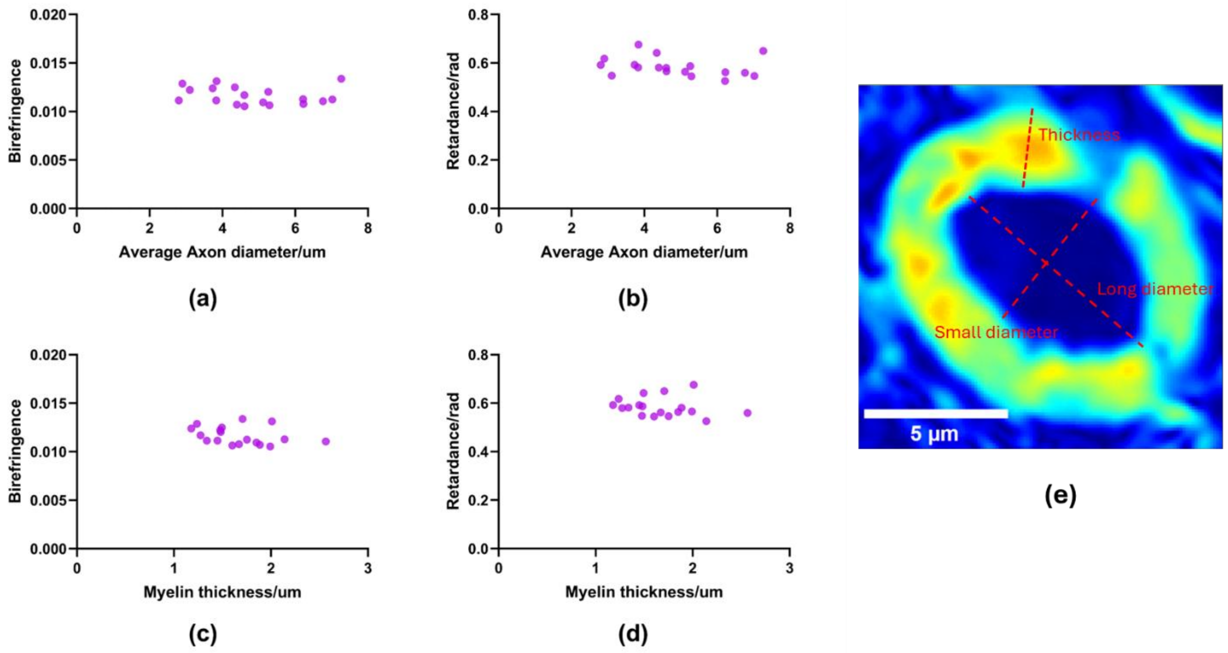

Fig. S5 Invariance of myelin optical properties across axonal geometries. (a–d) Scatter plots demonstrating that myelin birefringence and retardance are independent of axonal dimensions for the range indicated. Birefringence

shows no systematic correlation with average axon diameter (average between small diameter and large diameter in (e)) (a) or myelin thickness (c). Similarly, retardance (rad) remains consistent across varying diameters (b) and thicknesses (d), indicating that these optical properties are intrinsic to myelin composition rather than macro-morphology. (e) Representative retardance map of a myelinated axon cross-section (false-colored) showing quantified parameters: long diameter (major axis), short diameter (minor axis), and myelin thickness. Scale bar = 5 µm.
